# A Brief Review on Adult-Onset Coats’ Disease

**DOI:** 10.22336/rjo.2024.40

**Published:** 2024

**Authors:** Mary Stephen, Shreyas Temkar, Jayasri Periyandavan, Kalyan Basa

**Affiliations:** Department of Ophthalmology, JIPMER, Puducherry, India

**Keywords:** Coats’ disease, adult-onset, exudation, vision loss, anti-VEGF

## Abstract

Adult-onset Coats disease is an uncommon and vision-threatening disease characterized by the development of abnormal blood vessels in the retina. Coats’ disease commonly affects children in the first decade of life, but very rarely manifests in adults after the third decade of life, or who characteristically present with unilateral vision loss. Despite being a sight-threatening disease, the etiology remains inconclusive and various genetic and vascular abnormalities are implicated. Diagnosis relies on ophthalmologic examination, fundus photography, fluorescein angiography, and optical coherence tomography. Treatment modalities include laser photocoagulation, intravitreal injections of anti-vascular endothelial growth factor agents, and, in advanced cases, surgical interventions are needed and the treatment is aimed at avoiding complications like retinal detachment and neovascular glaucoma, which were comparatively rare in adult-onset Coats’ disease. Despite therapeutic advancements, the prognosis varies, with some patients experiencing significant visual impairment.

This review outlines the clinical features, diagnosis, management, and prognosis of adult-onset Coats’ disease, underscoring the importance of early detection and intervention in optimizing visual outcomes.

## Introduction

Coats’ disease was defined in 1908 by George Coats, a Scottish ophthalmologist, as retinal telangiectasia, exudation, and aneurysm in young boys. Etiopathogenesis, clinical presentation, and management of Coats’ disease have been studied in detail over the past decade and this review briefly describes the adult onset of Coats’ disease. The disease entity is characterized by intra or subretinal exudation primarily in the posterior pole and extensive retinal telangiectasia commonly in the retinal periphery. According to prospective research in the United Kingdom, the prevalence of Coats’ disease is 0.09 per 100,000 people and 85% of the affected patients were male; leukocoria or strabismus was the commonest presentation and about 44% of patients were blind at presentation and the disease entity was much more severe in paediatric patients than adult patients [1]. Hypercholesterolemia, hypertension, and diabetes have been linked to Coats’ disease in adults. However, no such link has been shown for childhood-onset disease. Adult Coats’ disease is an idiopathic retinal exudative vascular disease with distinct retinal characteristics compared to childhood disease. A report by Pulido et al. described Coats’ disease in a 30-year-old patient with extensive retinal exudation in one eye along with lipid deposition and described it as recurrent [2]. The lipid exudation noted in adult-onset Coats’ disease is almost similar to paediatric disease and the involved vessel is closer to the macula. Adults may experience vascular anomalies in peripheral and juxtamacular locations, including localized lipid deposition, bleeding around macroaneurysms, and delayed disease development. Smithen et al. described 13 patients with Coats’ disease with a mean age of onset of 50 years and differences noted from paediatric patients, including the slow rate of disease progression, confined area of involvement, and larger vascular dilatation with the area of adjacent haemorrhage [3].

## Pathophysiology of the Coats’ disease

Coats’ disease etiopathogenesis has been studied for the past few decades and a clear description is yet to be eluded and micro-vasculature anomalies are the primary factor leading to blood-retinal barrier breakdown with leakage of plasma and contents leading to extensive exudation. Loss of pericytes and degeneration of endothelial cells will result in aneurysm formation leading to exudation, which is rich in lipid and ischemia leading to retinal thickening and cyst formation. The exudation can be sub- or intra-retinal, and composed of various components including lipid-laden macrophages, which are periodic Acid Schiff positive and cholesterol clefts with altered vascular dynamics in the retro-bulbar area, especially in the later stage of disease [4]. Qi Zhao et al. studied retro-bulbar hemodynamic changes using color Doppler imaging. They found that the eye with Coats’ disease had decreased hemodynamic parameters compared to other normal eyes. They concluded that a detailed study is needed to make further conclusive evidence regarding hemodynamic changes and Coats’ disease [5]. Lim et al. showed that the retina of an enucleated eye with inflammatory Coats’ disease had perivascular T cell and macrophage infiltration and noted that immunopathologic features help better understand Coats’ disease [6]. Ghassemi et al. examined the protozoan and viral infections, as well as the serum hypercoagulability status, in 22 consecutive patients. Although these authors could not discover a connection between the variables and Coats’ disease, they found that children with the condition had higher serum levels of beta globulin [7]. Studies have revealed that aqueous humor samples from patients with progressively more advanced Coats’ illness had significantly higher nitric oxide levels and vascular endothelial growth factor (VEGF) [8]. The vasculature is the primary site of abnormalities in younger and older patients with Coats’ disease. One significant feature of the condition is capillary nonperfusion and remodeling of the surviving capillaries. The residual capillaries could get bigger, develop aneurysmal dilatation, and take on a filigree-like shape, which will leak, leading to exudation.

## Clinical manifestations

**Fig. 1:**
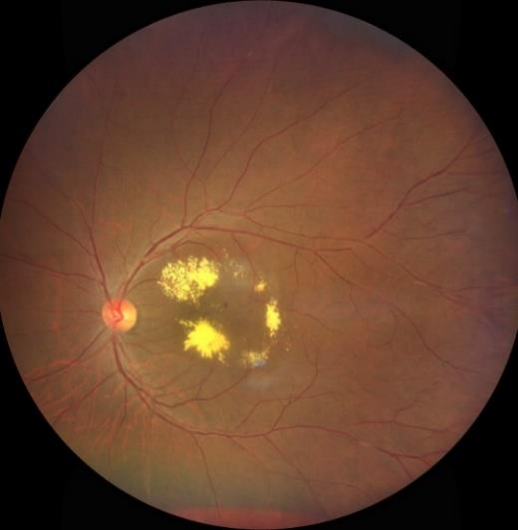
Color fundus photograph of patient showing dense exudation with thickening of the retina in the posterior pole

The classical Coats’ disease will manifest in young adult males with the age of onset around 6-8 years and the diagnosis in adulthood with a mean age of 35 years is rare [9]. Adults with Coats’ disease typically exhibit unilateral vascular telangiectasia, lipid exudation, microaneurysms and macroaneurysms, macular edema, areas of capillary nonperfusion with adjacent webs of filigree-like capillaries, and no retinal neovascularization (**Fig. 1**).

Adult Coats’ disease has two manifestations: one similar to Coats’ disease in children, and the other with retinal capillary anomalies. Coats’ disease-like manifestations can be observed in inflammation, infection, degenerative retinal changes, and after radiation therapy. Systemic diseases including diabetes mellitus, hypercholesterolemia, and hypertension have been associated with adult-onset disease and no such significant associations were noted in classical childhood disease [3]. This exudative retinal vascular disease of adulthood is often idiopathic, differing grossly from childhood disease including peripheral and localized deposition of lipids, macroaneurysms with haemorrhage due to rupture of aneurysm and slow progression of disease entity [10,11]. Rishi et al. studied 48 eyes with adult-onset Coats’ disease, finding that 21% of eyes had exudative retinal detachment, which is by far, much less than the study by Shield et al., who found that 81% of childhood Coats’ disease had exudative retinal detachment [12,13]. It also has been described that adult-onset Coats’ disease is less severe as less than 2 quadrants are involved in adults than diffuse involvement in childhood and so the end-stage complications including neovascular glaucoma, painful red eye, and phthisis bulbi are quite uncommon in adult-onset Coats’ disease [13-15]. The difference between childhood and adult-onset disease has been highlighted in **Table 1**.

**Table 1:** Difference between Childhood onset and Adult onset Coats’ disease

S. No.	Features	Childhood Coats’ disease	Adult onset Coats’ disease
1.	Age of onset	6-8 years	After 35 years
2.	Systemic association	No systemic association	Associated with Diabetes mellitus,Hypertension and Hypercholesterolemia
3.	Leukocoria	Common presentation	Not seen in adult-onset disease
4.	Rate of progression	Comparatively rapid progression	A slower rate of progression
5.	Exudative retinal detachment	High chance	Low chance
6.	Neovascular glaucoma	Comparatively high chance	Low chance
7.	Telangiectasia	Common	Rare
8.	Area of involvement	Macular and peripheral	Perimacular and peripheral
9.	Area of exudation	Diffuse	Focal
10.	Intra-retinal and pre-retinal haemorrhage	Rare	Common

## Investigations

The diagnosis of Coats’ disease needs detailed clinical evaluation and ophthalmoscopic examination and auxiliary tests are useful to aid the diagnosis.

B Scan ultrasound: The role of B-scan in adult-onset Coats’ disease is limited compared to childhood Coats’ disease as manifestations like hyperreflective exudates, exudative retinal detachment, and associated choroidal thickening are less common in adult-onset disease. Shields et al. described the color Doppler findings in Coats’ disease of childhood and adult-onset disease and described classical “Sand-like” change in cases associated with retinal detachment [16].

**Fig. 2:**
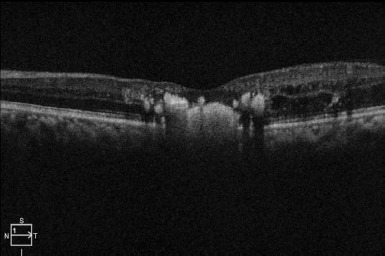
Optical Coherence Tomography image of patient showing altered foveal contour with disruption in RPE layer and IS-OS section and features suggestive of exudation in outer retinal layers

Optical Coherence Tomography: This imaging gives cross-sectional studies of the macula or around the interest area and helps in the quantification of the type as well as the extent of disease, and various other findings can be identified like subretinal/intraretinal exudates, subretinal fluid/nodules, macular hole/atrophy, ellipsoid zone disruption (**Fig. 2**).

However, the report from Mandhura et al. described the presence of only significant macular edema without the above-described findings and responded well to treatment [17]. Kang et al. studied Coats’ disease in 71 eyes with most adult-onset Coats’ disease. They found that Optical Coherence Tomography (OCT) is important in disease entity monitoring. Many cases in their study showed signs of resolution in wide-field imaging post-treatment and OCT showed persistent leakage leading to exudation [18].

OCT Angiography (OCT-A) has been described as beneficial in Childhood-onset Coats’ disease and in cases where performing Fundus Fluorescein Angiography is not possible, as OCT-A is non-invasive [19]. The role of OCT-A in adult-onset Coats’ disease is limited, as the literature is scarce and needs further research.

**Fig. 3:**
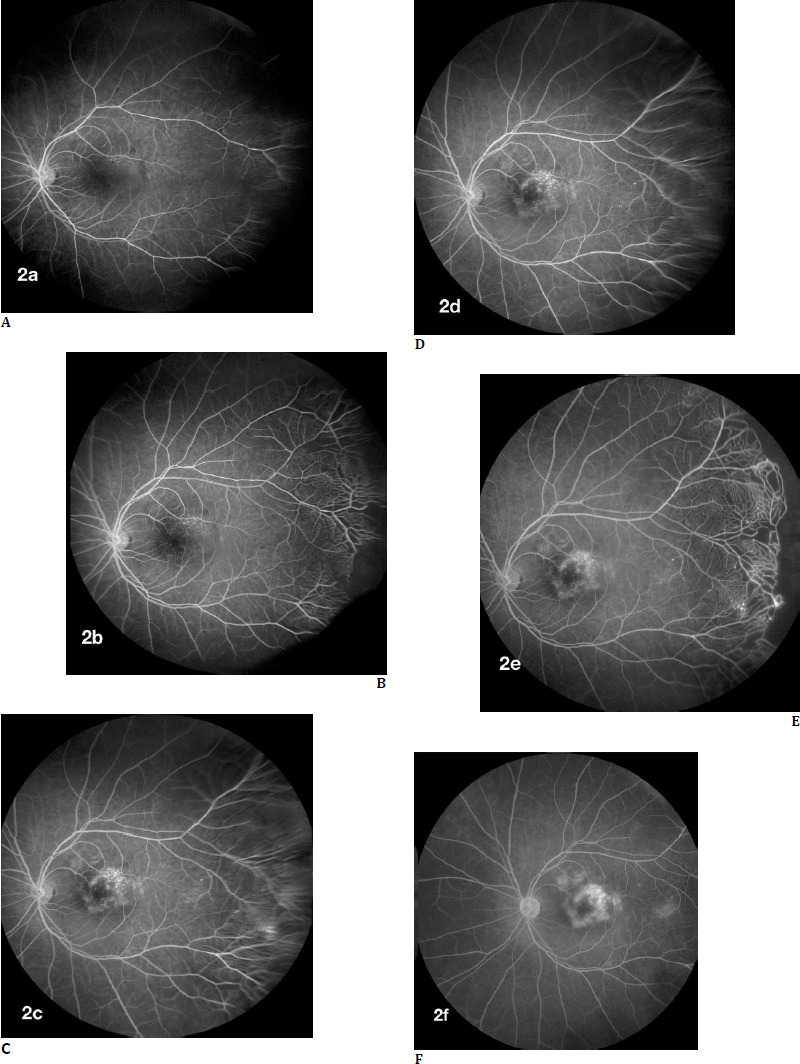
Fundus fluorescein angiography image showing a sequential increase in hyperfluorescence from **A** to **F**, and peripheral pruning of vessels noted with the area of avascularity

Fundus Fluorescein Angiography (FFA) is described as an important ancillary tool in diagnosing and identifying the classical findings in Coats’ disease like exudation, exudative retinal detachment, and retinal telangiectasia of light bulb configuration in the periphery are better appreciated [13]. In their case series, Kumar et al. described the importance of wide-field angiography in adult-onset Coats’ disease [20], where a patient with epiretinal membrane on evaluation had Coats’ disease with peripheral retinal telangiectasia, vascular leakage, and capillary nonperfusion areas. The study also revealed the presence of temporal periphery telangiectasia, avascular periphery, neo-vascularization in the periphery, and vascular leakage in adult patients (**Fig. 3 A-F**).

### Differential diagnosis

Paediatric Coats’ disease has all the differentials of Leukocoria and retinoblastoma, is the most significant one, and, in adult-onset disease, retinal detachment, cytomegalovirus or toxoplasma retinitis, and choroidal melanoma are close differentials to Coats’ disease.

### Treatment

Treatment aims to cause intra and sub-retinal exudates resolution in Coats’ disease, by eliminating the telangiectatic vessels. Adult onset Coats’ disease usually has good vision compared to paediatric Coats’ disease and the causes for vision loss described were macular edema/exudates, foveal ischemia, and the epiretinal membrane [21].

### Laser photocoagulation

Focal laser photocoagulation of the peripheral telangiectatic vessels is still a commonly performed treatment modality. It aims to ablate the telangiectatic vessel and reduce exudation and detachment, however, it depends on the location of the pathology. Nucci et al. reported a 10-year follow-up study of patients with Coats disease of various ages treated with selective laser photocoagulation without any other additional treatment despite the severity of the disease. The study found that young patients responded well, compared to adult patients, characterized by obliteration of telangiectatic vessels and absorption of exudation by four to eight weeks [22].

### Anti-Vascular Endothelial Growth Factor (VEGF)

The treatment trend shifted to anti-VEGF intravitreal injections from understanding the disease process and severity with its relation to VEGF. Qi Zhao found that VEGF concentration in the anterior chamber was significantly higher than in normal age-matched subjects compared to Coats’ disease and higher VEGF levels were noted based on the severity of the disease with a much higher level in advanced disease and posed a risk of exudative retinal detachment [23]. Recent studies also suggested the role of anti-VEGF intravitreal injection as an adjuvant treatment with conventional treatment, and that the chance of end-stage disease is minimized with the advent of anti-VEGF in paediatric and adult age group patients [24]. Various reports describe the effectiveness of Bevacizumab intravitreal injection as a stand-alone or combination treatment with laser photocoagulation of the peripheral telangiectatic lesion [25-27]. Goel et al. reported significant improvement in macular exudation in adult Coats’ patients following a single dose of intravitreal Bevacizumab coupled with peripheral laser photocoagulation [25]. Despite its widespread usage and efficacy reported in various studies, anti-VEGF injections pose a risk of development of traction resulting in retinal detachment and vitreoretinal fibrosis, which has been reported by Ramasubramaniam et al., in their study, and who advised cautious use of intravitreal Bevacizumab in Coats’ disease with exudative detachment in any age group [28]. Various reports described the usefulness of intravitreal Ranibizumab injection for the treatment of Coats’ disease in adults and most reports described it as an adjuvant to Laser photocoagulation, which is often needed post-injection for the resolution of abnormal telangiectatic vessels in the periphery [29,30]. Alsaggaf et al. reported the efficacy of Aflibercept intravitreal injection in adult-onset Coats’ disease patients, and, based on literature, established that aflibercept is more effective than other anti-VEGF, as the former targets both VEGF and the placental growth factor [31,32]. The use of Brolucizumab has been reported by Patel et al. in a 9-year-old patient with advanced Coats’ disease and found complete resolution with a single dose of injection; however, the use in adult-onset Coats’ disease has not been reported so far [33].

### Cryotherapy

Cryotherapy use has been described as a conventional treatment modality that deals with the peripheral telangiectatic vessels inaccessible to laser photocoagulation [3,12,17]. Most recent studies shifted the treatment options to anti-VEGF injection, followed by laser photocoagulation, and reported complete resolution of exudation.

### Vitrectomy

The need for pars plana vitrectomy in adult-onset Coats’ disease patients has been commonly described for macular complications like epiretinal membrane and full-thickness macular hole. Mino et al. reported the successful treatment of epiretinal membrane in adult-onset Coats’ disease with 25 Gauge pars plana vitrectomy with intraoperative laser photocoagulation and cryotherapy of peripheral abnormal vessels and good postoperative outcome [34]. Full-thickness macular Hole in adult-onset Coats’ disease is rarer than the epiretinal membrane and successful correction of the same with pars plana vitrectomy has been reported [35].

## Conclusion

Adult onset Coats’ disease is rare and the mean age of presentation is in the fourth decade with a comparatively less severe nature than paediatric Coats’ disease, where painful blind eye and neovascular glaucoma are observed. Association with systemic diseases like diabetes, hypertension, and hypercholesterolemia are often noted and warranted treatment for the same. Multi-modal imaging is helpful for diagnosis with detailed ophthalmoscopic examination and treatment options though varied, laser photocoagulate and anti-VEGF intravitreal injections are commonly used. Vitrectomy is reserved for cases with complications like epiretinal membrane and full-thickness macular hole.
